# HAV Infection Associated with Hemophagocytic Syndrome

**DOI:** 10.1155/2023/7799252

**Published:** 2023-01-10

**Authors:** H. Aamri, R. Elqadiry, H. Nassih, A. Bourrahouat, I. Ait Sab

**Affiliations:** ^1^Department of Pediatrics, Children's University Hospital, Marrakech, Morocco; ^2^Child Health and Development Research Unit, Cadi Ayyad University, Marrakech, Morocco

## Abstract

Hemophagocytic syndrome is a rare disease that can cause severe illness and death. This condition is caused by the presence of antibodies against the hepatitis A virus. A positive anti-IGM antibody was identified in our 7-year-old patient with severe hepatitis A. A week after the hepatitis A was diagnosed, the patient experienced pancytopenia, which was worsened by prolonged fever. He was then diagnosed with macrophage activation syndrome. The treatment with steroids improved the clinical and biological evolution of the condition.

## 1. Introduction

Hemophagocytic lymphohistiocytosis (HLH) is a rare existence-threatening syndrome which is characterized by activation of lymphocytes and histiocytes. It presents with high and prolonged fever, a maculopapular rash, hepatosplenomegaly, peripheral blood cytopenia, hyperferritinemia, coagulopathy, hyperlipidemia, and hemophagocytosis found in the bone marrow, lymph nodes. or spleen [1].

Secondary HLH is due specifically to underlying contamination, most of the infections triggering HLH, and viral infections are the most frequent, with Epstein–Barr virus (EBV), cytomegalovirus (CMV), human immunodeficiency virus (HIV), and human herpesvirus type 6 (HHV-6) [2].

HLH syndrome triggered by hepatitis A virus infection is not often encountered. Despite the frequency of hepatitis within the pediatric population, there are a few case reports approximately HAV-related HLH syndrome [2].

The main aim of this case report is to explain a case of an endemic hepatitis infection which triggered hemophagocytic lymphohistiocytosis syndrome.

## 2. Case Report

A 4-year-oldinfant boy with a history of soft tissue tuberculosis was treated at the age of one year. In November 2021, he was admitted to a university hospital with acute jaundice, fever, and vomiting. He was diagnosed with acute liver failure as a result of an infection with hepatitis A virus. The anti-HAV antibody immunoglobulin M (IgM) was elevated, and antibody immunoglobulin G (IgG) was negative. It was detected by using an enzyme-linked immunosorbent assay (ELISA). It was confirmed by the detection of HAV by real-time PCR. Serological tests for hepatitis B virus (HBV), hepatitis C virus (HCV), human immunodeficiency virus (HIV), cytomegalovirus (CMV), toxoplasmosis, rubella, and EBV were all negative. The infant still had a high fever, asthenia, and myalgia a week later. The patient had a painful case of hepatomegaly and splenomegaly. Following a thorough examination, the patient was diagnosed with sepsis. Despite supportive antibiotic treatment, he deteriorated with persistent fever and irritability. The liver was palpable at 10 cm with a hepatic arrow, but the rate was not. In addition to a macular rash and edema in the lower and upper extremities, the following laboratory results were obtained: hemoglobin 5.5 g/dL, mean corpuscular volume (MCV) 81.2 fL, white blood cell count (WBC) 0.62 103/*μ*L, and platelet count 97 103/*μ*L. Transaminase levels were found to be elevated in the initial laboratory examination (Table 1).

Urine cultures came back negative. However, the blood culture revealed the presence of two bacteria: *Leuconostoc citreum* and *Brevibacterium ravenspurgense*; he was treated with antibiotics, but the fever and asthenia persisted. An aspiration of bone marrow revealed the presence of histiocytes and lymphocytes at the medullary level. For three days, he received steroids via IV at a dose of 1 g/1.73 m^2^ and additional steroids via OS. On the third day of treatment, the patient's fever returned to normal, and his overall condition improved. On the fifth day of treatment, his ferritin level was reduced to 760 ng/L and his WBC level increased to 14400/mm^3^. His overall health had improved. The patient was discharged ten days later with no complaints. At the fourth month of follow-up, he was in good health.

## 3. Discussion

Hepatitis is an endemic disease that affects children in various ways, including asymptomatic condition, jaundice, and subicterus. Children under the age of six are asymptomatic and rarely present with a complex form. Any contamination caused by chronic hepatitis A should be investigated for complications, particularly fatal hematological damage, such as macrophagic activation syndrome, postinfectious thrombocytopenic purpura, or postcontamination hepatic aplasia. In comparison to other viruses, HLH is not a common complication of hepatitis A [3, 4].

Haemophagocytic syndrome is a rare but potentially fatal disease resulting from dysregulated activation and proliferation of lymphocytes. According to the Guidelines 2004, the diagnostic guidelines are as follows: 1/fever, 2/splenomegaly, 3/cytopenias affecting at least two of three lineages in the peripheral blood (affecting more than two cell lineages, haemoglobin ≤9 g/dL), 4/hypertriglyceridemia (triglycerides ≥265 mg/dL) and/or hypofibrinogenemia (fibrinogen ≤150 mg/dL), and 5/hemophagocytosis in the bone marrow, spleen, or lymph nodes, 6/low or absent NK-cell activity, 7/hyperferritinemia (ferritin ≥500 ng/mL), and 8/high levels of sIL-2r (interleukin-2R*α* chain ≥2400 IU/mL). Altogether, five of the eight criteria must be fulfilled, but patients with a molecular diagnosis consistent with HLH do not necessarily need to fulfill the diagnostic criteria [5].

Bay et al. described two case reviews about two pediatric patients without a clinical history of HAV-related macrophage activation syndrome. Each patient had been handled with intravenous immunoglobulin (IVIG) and accomplished remission. Our patient met 5 of the 8 standards of HLH (fever, splenomegaly, cytopenia, hypertriglyceridemia, and hyperferritinemia). Due to technical issues, interleukin-2 (il-2) ranges have not been evaluated in this example [5].

If left untreated, HLH is a deadly illness. According to the HLH-2014 therapy protocol, a preliminary treatment of dexamethasone and etoposide was administered for eight weeks. The therapy of HLH-associated HAV is not well characterized; however, some writers believe that IVIG is an effective treatment; alternative authors treat hemophagocytosis with steroids. We gave steroids to our patient, and he improved as a result [6].

Although HAV-associated HLH is uncommon, it should always be considered in the presence of a prolonged fever, a worsening in general condition, hepatosplenomegaly, and abnormal laboratory values [7].

## Figures and Tables

**Table 1:** Clinical and laboratory findings of the patient.

Criteria	Our patient
IgM hepatitis A	260 UI/mL
PCR for hepatitis A	+
Fever	+
Hepatomegaly	+
Splenomegaly	+
White cell count (5.0–11 × 103/L)	0, 62
Platelet count (150–400 × 103/L)	97
Hemoglobin (11.5–14 g/dL)	5.5
C-reactive protein (mg/L)	66
ALT (7–61 U/L)/AST (7–67 U/L)	2637/1448
Prothrombin time (12.6–14.2 s)/aPTT (35–45 s)	46%
Bilirubin (0.1–1 mg/dL)	—
Triglycerides (1.5–2.5 g/L)	4.6
Ferritin (7–142 ng/mL)	3480
Fibrinogen (1.5–4.0 g/L)	2.1
Natremia (136–145 mEq/L)	129
Hemophagocytosis in bone marrow	—
Levels of sIL-2r	Not done
NK-cell activity	Not done
